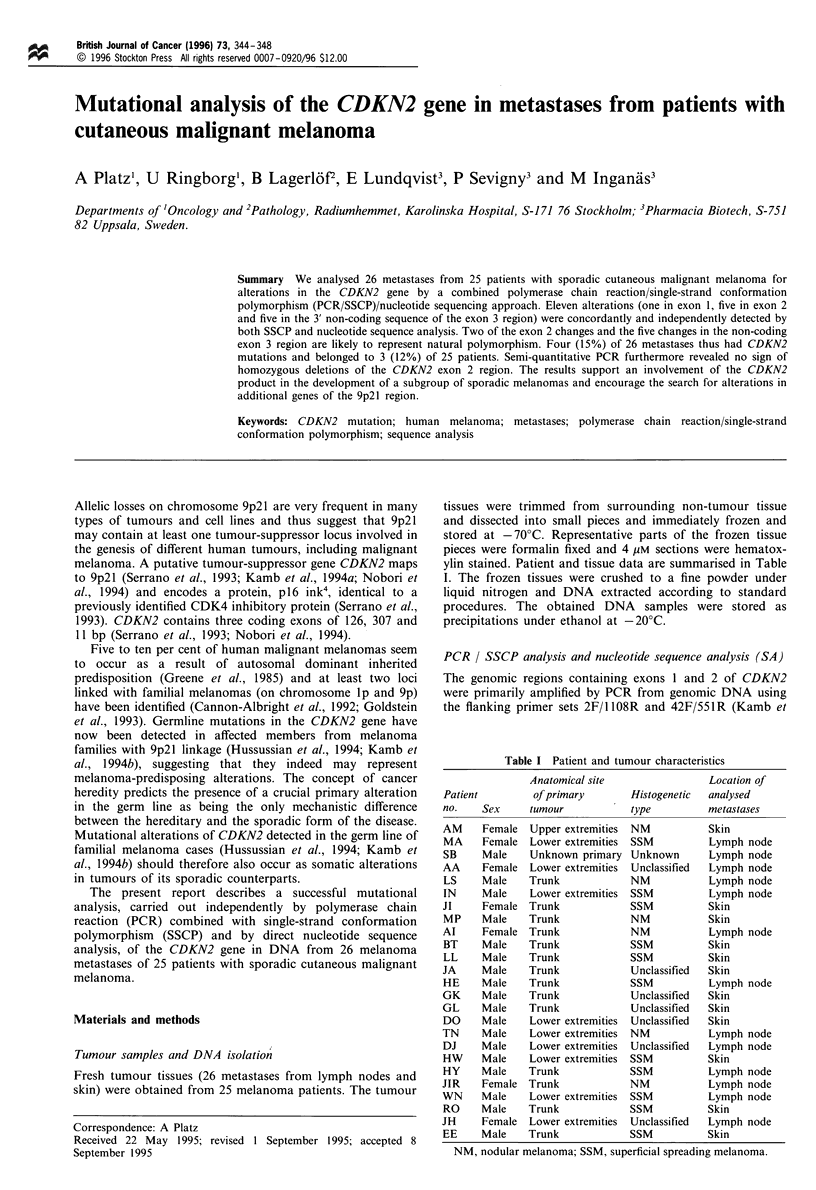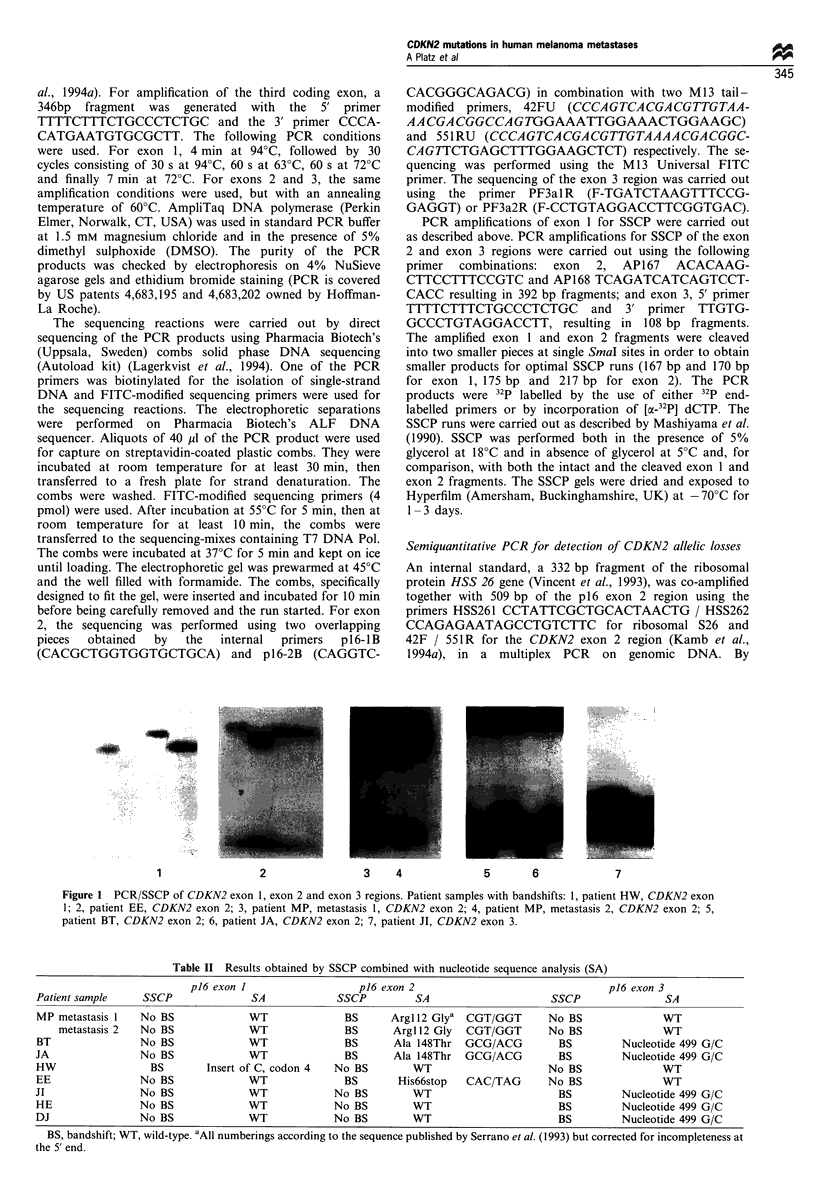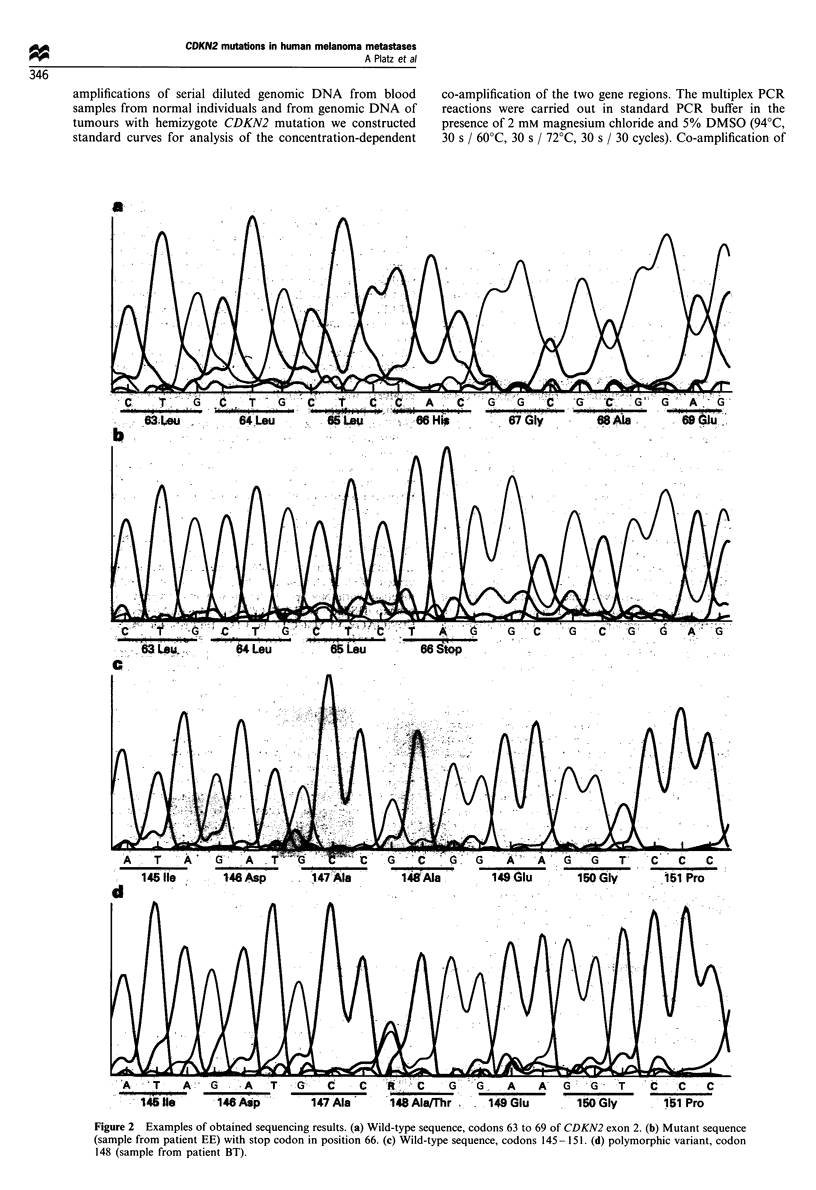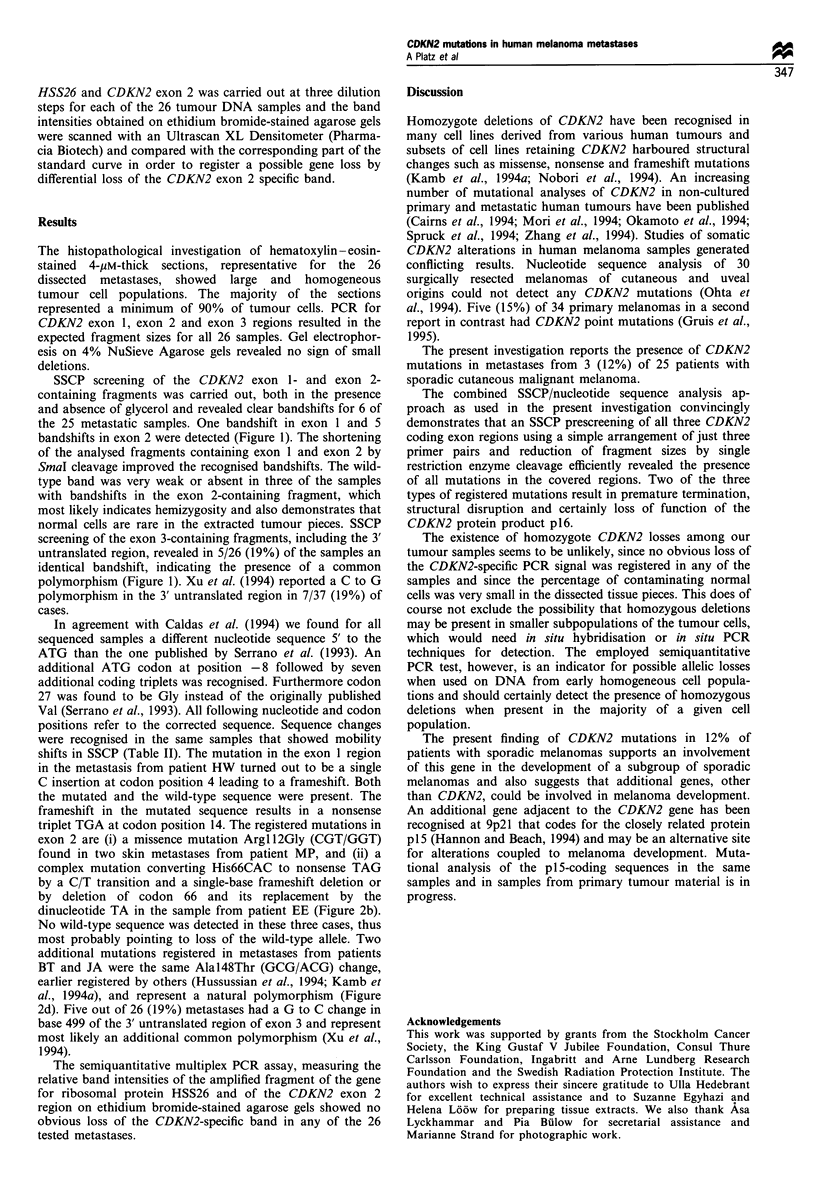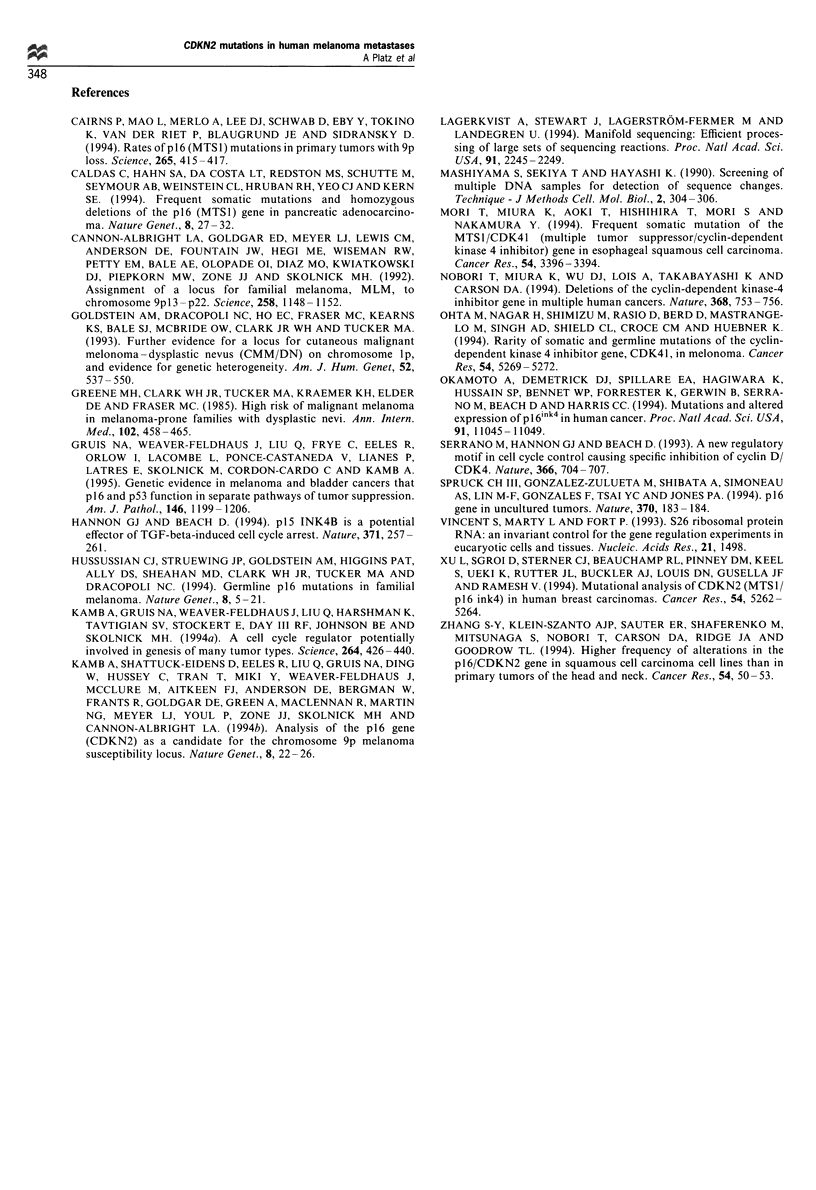# Mutational analysis of the CDKN2 gene in metastases from patients with cutaneous malignant melanoma.

**DOI:** 10.1038/bjc.1996.59

**Published:** 1996-02

**Authors:** A. Platz, U. Ringborg, B. Lagerlöf, E. Lundqvist, P. Sevigny, M. Inganäs

**Affiliations:** Department of Oncology, Radiumhemmet, Karolinska Hospital, Stockholm, Sweden.

## Abstract

**Images:**


British Journal of Cancer (1996) 73, 344–348
© 1996 Stockton Press All rights reserved 0007–0920/96 $12.00

### Mutational analysis of the *CDKN2* gene in metastases from patients with cutaneous malignant melanoma

A Platz[1], U Ringborg[1], B Lagerlöf[2], E Lundqvist[3], P Sevigny[3] and M Inganäs[3]

*Departments of [1]Oncology and [2]Pathology, Radiumhemmet, Karolinska Hospital, S-171 76 Stockholm; [3]Pharmacia Biotech, S-751 82 Uppsala, Sweden.*

**Summary** We analysed 26 metastases from 25 patients with sporadic cutaneous malignant melanoma for alterations in the *CDKN2* gene by a combined polymerase chain reaction/single-strand conformation polymorphism (PCR/SSCP)/nucleotide sequencing approach. Eleven alterations (one in exon 1, five in exon 2 and five in the 3′ non-coding sequence of the exon 3 region) were concordantly and independently detected by both SSCP and nucleotide sequence analysis. Two of the exon 2 changes and the five changes in the non-coding exon 3 region are likely to represent natural polymorphism. Four (15%) of 26 metastases thus had *CDKN2* mutations and belonged to 3 (12%) of 25 patients. Semi-quantitative PCR furthermore revealed no sign of homozygous deletions of the *CDKN2* exon 2 region. The results support an involvement of the *CDKN2* product in the development of a subgroup of sporadic melanomas and encourage the search for alterations in additional genes of the 9p21 region.

**Keywords:** *CDKN2* mutation; human melanoma; metastases; polymerase chain reaction/single-strand conformation polymorphism; sequence analysis

Allelic losses on chromosome 9p21 are very frequent in many types of tumours and cell lines and thus suggest that 9p21 may contain at least one tumour-suppressor locus involved in the genesis of different human tumours, including malignant melanoma. A putative tumour-suppressor gene *CDKN2* maps to 9p21 (Serrano *et al.*, 1993; Kamb *et al.*, 1994*a*; Nobori *et al.*, 1994) and encodes a protein, p16 ink[4], identical to a previously identified CDK4 inhibitory protein (Serrano *et al.*, 1993). *CDKN2* contains three coding exons of 126, 307 and 11 bp (Serrano *et al.*, 1993; Nobori *et al.*, 1994).

Five to ten per cent of human malignant melanomas seem to occur as a result of autosomal dominant inherited predisposition (Greene *et al.*, 1985) and at least two loci linked with familial melanomas (on chromosome 1p and 9p) have been identified (Cannon-Albright *et al.*, 1992; Goldstein *et al.*, 1993). Germline mutations in the *CDKN2* gene have now been detected in affected members from melanoma families with 9p21 linkage (Hussussian *et al.*, 1994; Kamb *et al.*, 1994*b*), suggesting that they indeed may represent melanoma-predisposing alterations. The concept of cancer heredity predicts the presence of a crucial primary alteration in the germ line as being the only mechanistic difference between the hereditary and the sporadic form of the disease. Mutational alterations of *CDKN2* detected in the germ line of familial melanoma cases (Hussussian *et al.*, 1994; Kamb *et al.*, 1994*b*) should therefore also occur as somatic alterations in tumours of its sporadic counterparts.

The present report describes a successful mutational analysis, carried out independently by polymerase chain reaction (PCR) combined with single-strand conformation polymorphism (SSCP) and by direct nucleotide sequence analysis, of the *CDKN2* gene in DNA from 26 melanoma metastases of 25 patients with sporadic cutaneous malignant melanoma.

### Materials and methods

### *Tumour samples and DNA isolation*

Fresh tumour tissues (26 metastases from lymph nodes and skin) were obtained from 25 melanoma patients. The tumour tissues were trimmed from surrounding non-tumour tissue and dissected into small pieces and immediately frozen and stored at −70°C. Representative parts of the frozen tissue pieces were formalin fixed and 4 μM sections were hematoxylin stained. Patient and tissue data are summarised in Table I. The frozen tissues were crushed to a fine powder under liquid nitrogen and DNA extracted according to standard procedures. The obtained DNA samples were stored as precipitations under ethanol at −20°C.

### *PCR / SSCP analysis and nucleotide sequence analysis (SA)*

The genomic regions containing exons 1 and 2 of *CDKN2* were primarily amplified by PCR from genomic DNA using the flanking primer sets 2F/1108R and 42F/551R (Kamb *et*

**Table I** Patient and tumour characteristics

| *Patient no.* | *Sex* | *Anatomical site of primary tumour* | *Histogenetic type* | *Location of analysed metastases* |
|---|---|---|---|---|
| AM | Female | Upper extremities | NM | Skin |
| MA | Female | Lower extremities | SSM | Lymph node |
| SB | Male | Unknown primary | Unknown | Lymph node |
| AA | Female | Lower extremities | Unclassified | Lymph node |
| LS | Male | Trunk | NM | Lymph node |
| IN | Male | Lower extremities | SSM | Lymph node |
| JI | Female | Trunk | SSM | Skin |
| MP | Male | Trunk | NM | Skin |
| AI | Female | Trunk | NM | Lymph node |
| BT | Male | Trunk | SSM | Skin |
| LL | Male | Trunk | SSM | Skin |
| JA | Male | Trunk | Unclassified | Skin |
| HE | Male | Trunk | SSM | Lymph node |
| GK | Male | Trunk | Unclassified | Skin |
| GL | Male | Trunk | Unclassified | Skin |
| DO | Male | Lower extremities | Unclassified | Skin |
| TN | Male | Lower extremities | NM | Lymph node |
| DJ | Male | Lower extremities | Unclassified | Lymph node |
| HW | Male | Lower extremities | SSM | Skin |
| HY | Male | Trunk | SSM | Lymph node |
| JIR | Female | Trunk | NM | Lymph node |
| WN | Male | Lower extremities | SSM | Lymph node |
| RO | Male | Trunk | SSM | Skin |
| JH | Female | Lower extremities | Unclassified | Lymph node |
| EE | Male | Trunk | SSM | Skin |

NM, nodular melanoma; SSM, superficial spreading melanoma.

Correspondence: A Platz
Received 22 May 1995; revised 1 September 1995; accepted 8 September 1995

*al*., 1994*a*). For amplification of the third coding exon, a 346bp fragment was generated with the 5′ primer TTTTCTTTCTGCCCTCTGC and the 3′ primer CCCA-CATGAATGTGCGCTT. The following PCR conditions were used. For exon 1, 4 min at 94°C, followed by 30 cycles consisting of 30 s at 94°C, 60 s at 63°C, 60 s at 72°C and finally 7 min at 72°C. For exons 2 and 3, the same amplification conditions were used, but with an annealing temperature of 60°C. AmpliTaq DNA polymerase (Perkin Elmer, Norwalk, CT, USA) was used in standard PCR buffer at 1.5 mM magnesium chloride and in the presence of 5% dimethyl sulphoxide (DMSO). The purity of the PCR products was checked by electrophoresis on 4% NuSieve agarose gels and ethidium bromide staining (PCR is covered by US patents 4,683,195 and 4,683,202 owned by Hoffman-La Roche).

The sequencing reactions were carried out by direct sequencing of the PCR products using Pharmacia Biotech's (Uppsala, Sweden) combs solid phase DNA sequencing (Autoload kit) (Lagerkvist *et al*., 1994). One of the PCR primers was biotinylated for the isolation of single-strand DNA and FITC-modified sequencing primers were used for the sequencing reactions. The electrophoretic separations were performed on Pharmacia Biotech's ALF DNA sequencer. Aliquots of 40 μl of the PCR product were used for capture on streptavidin-coated plastic combs. They were incubated at room temperature for at least 30 min, then transferred to a fresh plate for strand denaturation. The combs were washed. FITC-modified sequencing primers (4 pmol) were used. After incubation at 55°C for 5 min, then at room temperature for at least 10 min, the combs were transferred to the sequencing-mixes containing T7 DNA Pol. The combs were incubated at 37°C for 5 min and kept on ice until loading. The electrophoretic gel was prewarmed at 45°C and the well filled with formamide. The combs, specifically designed to fit the gel, were inserted and incubated for 10 min before being carefully removed and the run started. For exon 2, the sequencing was performed using two overlapping pieces obtained by the internal primers p16-1B (CACGCTGGTGGTGCTGCA) and p16-2B (CAGGTC-CACGGGCAGACG) in combination with two M13 tail-modified primers, 42FU (*CCCAGTCACGACGTTGTAA-AACGACGGCCAGT*GGAAATTGGAAACTGGAAGC) and 551RU (*CCCAGTCACGACGTTGTAAAACGACGGC-CAGT*TCTGAGCTTTGGAAGCTCT) respectively. The sequencing was performed using the M13 Universal FITC primer. The sequencing of the exon 3 region was carried out using the primer PF3a1R (F-TGATCTAAGTTTCCG-GAGGT) or PF3a2R (F-CCTGTAGGACCTTCGGTGAC).

PCR amplifications of exon 1 for SSCP were carried out as described above. PCR amplifications for SSCP of the exon 2 and exon 3 regions were carried out using the following primer combinations: exon 2, AP167 ACACAAG-CTTCCTTTCCGTC and AP168 TCAGATCATCAGTCCT-CACC resulting in 392 bp fragments; and exon 3, 5′ primer TTTTCTTTCTGCCCTCTGC and 3′ primer TTGTG-GCCCTGTAGGACCTT, resulting in 108 bp fragments. The amplified exon 1 and exon 2 fragments were cleaved into two smaller pieces at single *Sma*I sites in order to obtain smaller products for optimal SSCP runs (167 bp and 170 bp for exon 1, 175 bp and 217 bp for exon 2). The PCR products were $^{32}P$ labelled by the use of either $^{32}P$ end-labelled primers or by incorporation of [$\alpha$-$^{32}P$] dCTP. The SSCP runs were carried out as described by Mashiyama *et al*. (1990). SSCP was performed both in the presence of 5% glycerol at 18°C and in absence of glycerol at 5°C and, for comparison, with both the intact and the cleaved exon 1 and exon 2 fragments. The SSCP gels were dried and exposed to Hyperfilm (Amersham, Buckinghamshire, UK) at −70°C for 1–3 days.

### *Semiquantitative PCR for detection of CDKN2 allelic losses*

An internal standard, a 332 bp fragment of the ribosomal protein *HSS 26* gene (Vincent *et al*., 1993), was co-amplified together with 509 bp of the p16 exon 2 region using the primers HSS261 CCTATTCGCTGCACTAACTG / HSS262 CCAGAGAATAGCCTGTCTTC for ribosomal S26 and 42F / 551R for the *CDKN2* exon 2 region (Kamb *et al*., 1994*a*), in a multiplex PCR on genomic DNA. By

**Figure 1** PCR/SSCP of *CDKN2* exon 1, exon 2 and exon 3 regions. Patient samples with bandshifts: 1, patient HW, *CDKN2* exon 1; 2, patient EE, *CDKN2* exon 2; 3, patient MP, metastasis 1, *CDKN2* exon 2; 4, patient MP, metastasis 2, *CDKN2* exon 2; 5, patient BT, *CDKN2* exon 2; 6, patient JA, *CDKN2* exon 2; 7, patient JI, *CDKN2* exon 3.

**Table II** Results obtained by SSCP combined with nucleotide sequence analysis (SA)

| Patient sample | *p16 exon 1* SSCP | *p16 exon 1* SA | *p16 exon 2* SSCP | *p16 exon 2* SA | | *p16 exon 3* SSCP | *p16 exon 3* SA |
|---|---|---|---|---|---|---|---|
| MP metastasis 1 | No BS | WT | BS | Arg112 Gly[a] | CGT/GGT | No BS | WT |
| metastasis 2 | No BS | WT | BS | Arg112 Gly | CGT/GGT | No BS | WT |
| BT | No BS | WT | BS | Ala 148Thr | GCG/ACG | BS | Nucleotide 499 G/C |
| JA | No BS | WT | BS | Ala 148Thr | GCG/ACG | BS | Nucleotide 499 G/C |
| HW | BS | Insert of C, codon 4 | No BS | WT | | No BS | WT |
| EE | No BS | WT | BS | His66stop | CAC/TAG | No BS | WT |
| JI | No BS | WT | No BS | WT | | BS | Nucleotide 499 G/C |
| HE | No BS | WT | No BS | WT | | BS | Nucleotide 499 G/C |
| DJ | No BS | WT | No BS | WT | | BS | Nucleotide 499 G/C |

BS, bandshift; WT, wild-type. [a]All numberings according to the sequence published by Serrano *et al*. (1993) but corrected for incompleteness at the 5′ end.

amplifications of serial diluted genomic DNA from blood samples from normal individuals and from genomic DNA of tumours with hemizygote *CDKN2* mutation we constructed standard curves for analysis of the concentration-dependent co-amplification of the two gene regions. The multiplex PCR reactions were carried out in standard PCR buffer in the presence of 2 mM magnesium chloride and 5% DMSO (94°C, 30 s / 60°C, 30 s / 72°C, 30 s / 30 cycles). Co-amplification of

**Figure 2** Examples of obtained sequencing results. (**a**) Wild-type sequence, codons 63 to 69 of *CDKN2* exon 2. (**b**) Mutant sequence (sample from patient EE) with stop codon in position 66. (**c**) Wild-type sequence, codons 145–151. (**d**) polymorphic variant, codon 148 (sample from patient BT).

*HSS26* and *CDKN2* exon 2 was carried out at three dilution steps for each of the 26 tumour DNA samples and the band intensities obtained on ethidium bromide-stained agarose gels were scanned with an Ultrascan XL Densitometer (Pharmacia Biotech) and compared with the corresponding part of the standard curve in order to register a possible gene loss by differential loss of the *CDKN2* exon 2 specific band.

### Results

The histopathological investigation of hematoxylin–eosin-stained 4-μM-thick sections, representative for the 26 dissected metastases, showed large and homogeneous tumour cell populations. The majority of the sections represented a minimum of 90% of tumour cells. PCR for *CDKN2* exon 1, exon 2 and exon 3 regions resulted in the expected fragment sizes for all 26 samples. Gel electrophoresis on 4% NuSieve Agarose gels revealed no sign of small deletions.

SSCP screening of the *CDKN2* exon 1- and exon 2-containing fragments was carried out, both in the presence and absence of glycerol and revealed clear bandshifts for 6 of the 25 metastatic samples. One bandshift in exon 1 and 5 bandshifts in exon 2 were detected (Figure 1). The shortening of the analysed fragments containing exon 1 and exon 2 by *Sma*I cleavage improved the recognised bandshifts. The wild-type band was very weak or absent in three of the samples with bandshifts in the exon 2-containing fragment, which most likely indicates hemizygosity and also demonstrates that normal cells are rare in the extracted tumour pieces. SSCP screening of the exon 3-containing fragments, including the 3′ untranslated region, revealed in 5/26 (19%) of the samples an identical bandshift, indicating the presence of a common polymorphism (Figure 1). Xu *et al.* (1994) reported a C to G polymorphism in the 3′ untranslated region in 7/37 (19%) of cases.

In agreement with Caldas *et al.* (1994) we found for all sequenced samples a different nucleotide sequence 5′ to the ATG than the one published by Serrano *et al.* (1993). An additional ATG codon at position −8 followed by seven additional coding triplets was recognised. Furthermore codon 27 was found to be Gly instead of the originally published Val (Serrano *et al.*, 1993). All following nucleotide and codon positions refer to the corrected sequence. Sequence changes were recognised in the same samples that showed mobility shifts in SSCP (Table II). The mutation in the exon 1 region in the metastasis from patient HW turned out to be a single C insertion at codon position 4 leading to a frameshift. Both the mutated and the wild-type sequence were present. The frameshift in the mutated sequence results in a nonsense triplet TGA at codon position 14. The registered mutations in exon 2 are (i) a missence mutation Arg112Gly (CGT/GGT) found in two skin metastases from patient MP, and (ii) a complex mutation converting His66CAC to nonsense TAG by a C/T transition and a single-base frameshift deletion or by deletion of codon 66 and its replacement by the dinucleotide TA in the sample from patient EE (Figure 2b). No wild-type sequence was detected in these three cases, thus most probably pointing to loss of the wild-type allele. Two additional mutations registered in metastases from patients BT and JA were the same Ala148Thr (GCG/ACG) change, earlier registered by others (Hussussian *et al.*, 1994; Kamb *et al.*, 1994*a*), and represent a natural polymorphism (Figure 2d). Five out of 26 (19%) metastases had a G to C change in base 499 of the 3′ untranslated region of exon 3 and represent most likely an additional common polymorphism (Xu *et al.*, 1994).

The semiquantitative multiplex PCR assay, measuring the relative band intensities of the amplified fragment of the gene for ribosomal protein HSS26 and of the *CDKN2* exon 2 region on ethidium bromide-stained agarose gels showed no obvious loss of the *CDKN2*-specific band in any of the 26 tested metastases.

### Discussion

Homozygote deletions of *CDKN2* have been recognised in many cell lines derived from various human tumours and subsets of cell lines retaining *CDKN2* harboured structural changes such as missense, nonsense and frameshift mutations (Kamb *et al.*, 1994*a*; Nobori *et al.*, 1994). An increasing number of mutational analyses of *CDKN2* in non-cultured primary and metastatic human tumours have been published (Cairns *et al.*, 1994; Mori *et al.*, 1994; Okamoto *et al.*, 1994; Spruck *et al.*, 1994; Zhang *et al.*, 1994). Studies of somatic *CDKN2* alterations in human melanoma samples generated conflicting results. Nucleotide sequence analysis of 30 surgically resected melanomas of cutaneous and uveal origins could not detect any *CDKN2* mutations (Ohta *et al.*, 1994). Five (15%) of 34 primary melanomas in a second report in contrast had *CDKN2* point mutations (Gruis *et al.*, 1995).

The present investigation reports the presence of *CDKN2* mutations in metastases from 3 (12%) of 25 patients with sporadic cutaneous malignant melanoma.

The combined SSCP/nucleotide sequence analysis approach as used in the present investigation convincingly demonstrates that an SSCP prescreening of all three *CDKN2* coding exon regions using a simple arrangement of just three primer pairs and reduction of fragment sizes by single restriction enzyme cleavage efficiently revealed the presence of all mutations in the covered regions. Two of the three types of registered mutations result in premature termination, structural disruption and certainly loss of function of the *CDKN2* protein product p16.

The existence of homozygote *CDKN2* losses among our tumour samples seems to be unlikely, since no obvious loss of the *CDKN2*-specific PCR signal was registered in any of the samples and since the percentage of contaminating normal cells was very small in the dissected tissue pieces. This does of course not exclude the possibility that homozygous deletions may be present in smaller subpopulations of the tumour cells, which would need *in situ* hybridisation or *in situ* PCR techniques for detection. The employed semiquantitative PCR test, however, is an indicator for possible allelic losses when used on DNA from early homogeneous cell populations and should certainly detect the presence of homozygous deletions when present in the majority of a given cell population.

The present finding of *CDKN2* mutations in 12% of patients with sporadic melanomas supports an involvement of this gene in the development of a subgroup of sporadic melanomas and also suggests that additional genes, other than *CDKN2*, could be involved in melanoma development. An additional gene adjacent to the *CDKN2* gene has been recognised at 9p21 that codes for the closely related protein p15 (Hannon and Beach, 1994) and may be an alternative site for alterations coupled to melanoma development. Mutational analysis of the p15-coding sequences in the same samples and in samples from primary tumour material is in progress.

**Acknowledgements**
This work was supported by grants from the Stockholm Cancer Society, the King Gustaf V Jubilee Foundation, Consul Thure Carlsson Foundation, Ingabritt and Arne Lundberg Research Foundation and the Swedish Radiation Protection Institute. The authors wish to express their sincere gratitude to Ulla Hedebrant for excellent technical assistance and to Suzanne Egyhazi and Helena Lööw for preparing tissue extracts. We also thank Åsa Lyckhammar and Pia Bülow for secretarial assistance and Marianne Strand for photographic work.

### References

CAIRNS P, MAO L, MERLO A, LEE DJ, SCHWAB D, EBY Y, TOKINO K, VAN DER RIET P, BLAUGRUND JE AND SIDRANSKY D. (1994). Rates of p16 (MTS1) mutations in primary tumors with 9p loss. *Science*, **265**, 415-417.

CALDAS C, HAHN SA, DA COSTA LT, REDSTON MS, SCHUTTE M, SEYMOUR AB, WEINSTEIN CL, HRUBAN RH, YEO CJ AND KERN SE. (1994). Frequent somatic mutations and homozygous deletions of the p16 (MTS1) gene in pancreatic adenocarcinoma. *Nature Genet.*, **8**, 27-32.

CANNON-ALBRIGHT LA, GOLDGAR ED, MEYER LJ, LEWIS CM, ANDERSON DE, FOUNTAIN JW, HEGI ME, WISEMAN RW, PETTY EM, BALE AE, OLOPADE OI, DIAZ MO, KWIATKOWSKI DJ, PIEPKORN MW, ZONE JJ AND SKOLNICK MH. (1992). Assignment of a locus for familial melanoma, MLM, to chromosome 9p13-p22. *Science*, **258**, 1148-1152.

GOLDSTEIN AM, DRACOPOLI NC, HO EC, FRASER MC, KEARNS KS, BALE SJ, MCBRIDE OW, CLARK JR WH AND TUCKER MA. (1993). Further evidence for a locus for cutaneous malignant melonoma-dysplastic nevus (CMM/DN) on chromosome 1p, and evidence for genetic heterogeneity. *Am. J. Hum. Genet*, **52**, 537-550.

GREENE MH, CLARK WH JR, TUCKER MA, KRAEMER KH, ELDER DE AND FRASER MC. (1985). High risk of malignant melanoma in melanoma-prone families with dysplastic nevi. *Ann. Intern. Med.*, **102**, 458-465.

GRUIS NA, WEAVER-FELDHAUS J, LIU Q, FRYE C, EELES R, ORLOW I, LACOMBE L, PONCE-CASTANEDA V, LIANES P, LATRES E, SKOLNICK M, CORDON-CARDO C AND KAMB A. (1995). Genetic evidence in melanoma and bladder cancers that p16 and p53 function in separate pathways of tumor suppression. *Am. J. Pathol.*, **146**, 1199-1206.

HANNON GJ AND BEACH D. (1994). p15 INK4B is a potential effector of TGF-beta-induced cell cycle arrest. *Nature*, **371**, 257-261.

HUSSUSSIAN CJ, STRUEWING JP, GOLDSTEIN AM, HIGGINS PAT, ALLY DS, SHEAHAN MD, CLARK WH JR, TUCKER MA AND DRACOPOLI NC. (1994). Germline p16 mutations in familial melanoma. *Nature Genet.*, **8**, 5-21.

KAMB A, GRUIS NA, WEAVER-FELDHAUS J, LIU Q, HARSHMAN K, TAVTIGIAN SV, STOCKERT E, DAY III RF, JOHNSON BE AND SKOLNICK MH. (1994*a*). A cell cycle regulator potentially involved in genesis of many tumor types. *Science*, **264**, 426-440.

KAMB A, SHATTUCK-EIDENS D, EELES R, LIU Q, GRUIS NA, DING W, HUSSEY C, TRAN T, MIKI Y, WEAVER-FELDHAUS J, MCCLURE M, AITKEEN FJ, ANDERSON DE, BERGMAN W, FRANTS R, GOLDGAR DE, GREEN A, MACLENNAN R, MARTIN NG, MEYER LJ, YOUL P, ZONE JJ, SKOLNICK MH AND CANNON-ALBRIGHT LA. (1994*b*). Analysis of the p16 gene (CDKN2) as a candidate for the chromosome 9p melanoma susceptibility locus. *Nature Genet.*, **8**, 22-26.

LAGERKVIST A, STEWART J, LAGERSTRÖM-FERMER M AND LANDEGREN U. (1994). Manifold sequencing: Efficient processing of large sets of sequencing reactions. *Proc. Natl Acad. Sci. USA*, **91**, 2245-2249.

MASHIYAMA S, SEKIYA T AND HAYASHI K. (1990). Screening of multiple DNA samples for detection of sequence changes. *Technique - J Methods Cell. Mol. Biol.*, **2**, 304-306.

MORI T, MIURA K, AOKI T, HISHIHIRA T, MORI S AND NAKAMURA Y. (1994). Frequent somatic mutation of the MTS1/CDK41 (multiple tumor suppressor/cyclin-dependent kinase 4 inhibitor) gene in esophageal squamous cell carcinoma. *Cancer Res.*, **54**, 3396-3394.

NOBORI T, MIURA K, WU DJ, LOIS A, TAKABAYASHI K AND CARSON DA. (1994). Deletions of the cyclin-dependent kinase-4 inhibitor gene in multiple human cancers. *Nature*, **368**, 753-756.

OHTA M, NAGAR H, SHIMIZU M, RASIO D, BERD D, MASTRANGELO M, SINGH AD, SHIELD CL, CROCE CM AND HUEBNER K. (1994). Rarity of somatic and germline mutations of the cyclin-dependent kinase 4 inhibitor gene, CDK41, in melonoma. *Cancer Res*, **54**, 5269-5272.

OKAMOTO A, DEMETRICK DJ, SPILLARE EA, HAGIWARA K, HUSSAIN SP, BENNET WP, FORRESTER K, GERWIN B, SERRANO M, BEACH D AND HARRIS CC. (1994). Mutations and altered expression of $p16^{ink4}$ in human cancer. *Proc. Natl Acad. Sci. USA*, **91**, 11045-11049.

SERRANO M, HANNON GJ AND BEACH D. (1993). A new regulatory motif in cell cycle control causing specific inhibition of cyclin D/CDK4. *Nature*, **366**, 704-707.

SPRUCK CH III, GONZALEZ-ZULUETA M, SHIBATA A, SIMONEAU AS, LIN M-F, GONZALES F, TSAI YC AND JONES PA. (1994). p16 gene in uncultured tumors. *Nature*, **370**, 183-184.

VINCENT S, MARTY L AND FORT P. (1993). S26 ribosomal protein RNA: an invariant control for the gene regulation experiments in eucaryotic cells and tissues. *Nucleic. Acids Res.*, **21**, 1498.

XU L, SGROI D, STERNER CJ, BEAUCHAMP RL, PINNEY DM, KEEL S, UEKI K, RUTTER JL, BUCKLER AJ, LOUIS DN, GUSELLA JF AND RAMESH V. (1994). Mutational analysis of CDKN2 (MTS1/p16 ink4) in human breast carcinomas. *Cancer Res.*, **54**, 5262-5264.

ZHANG S-Y, KLEIN-SZANTO AJP, SAUTER ER, SHAFERENKO M, MITSUNAGA S, NOBORI T, CARSON DA, RIDGE JA AND GOODROW TL. (1994). Higher frequency of alterations in the p16/CDKN2 gene in squamous cell carcinoma cell lines than in primary tumors of the head and neck. *Cancer Res.*, **54**, 50-53.